# Advances in Multimodality Cardiovascular Imaging in the Diagnosis of Heart Failure With Preserved Ejection Fraction

**DOI:** 10.3389/fcvm.2022.758975

**Published:** 2022-03-09

**Authors:** Alberico Del Torto, Andrea Igoren Guaricci, Francesca Pomarico, Marco Guglielmo, Laura Fusini, Francesco Monitillo, Daniela Santoro, Monica Vannini, Alexia Rossi, Giuseppe Muscogiuri, Andrea Baggiano, Gianluca Pontone

**Affiliations:** ^1^Department of Emergency and Acute Cardiac Care, Centro Cardiologico Monzino IRCCS, Milan, Italy; ^2^University Cardiology Unit, Policlinic University Hospital, Bari, Italy; ^3^Cardiovascular Imaging Department, Centro Cardiologico Monzino IRCCS, Milan, Italy; ^4^Department of Nuclear Medicine, University Hospital Zurich, Zurich, Switzerland; ^5^Department of Radiology, IRCCS Istituto Auxologico Italiano, San Luca Hospital, Milan, Italy; ^6^University Milano Bicocca, Milan, Italy

**Keywords:** HFpEF, heart failure, multimodality imaging, diastolic function, echocardiography, cardiovascular magnetic resonance, nuclear imaging, cardiovascular computed tomography

## Abstract

Heart failure with preserved ejection fraction (HFpEF) is a syndrome defined by the presence of heart failure symptoms and increased levels of circulating natriuretic peptide (NP) in patients with preserved left ventricular ejection fraction and various degrees of diastolic dysfunction (DD). HFpEF is a complex condition that encompasses a wide range of different etiologies. Cardiovascular imaging plays a pivotal role in diagnosing HFpEF, in identifying specific underlying etiologies, in prognostic stratification, and in therapeutic individualization. Echocardiography is the first line imaging modality with its wide availability; it has high spatial and temporal resolution and can reliably assess systolic and diastolic function. Cardiovascular magnetic resonance (CMR) is the gold standard for cardiac morphology and function assessment, and has superior contrast resolution to look in depth into tissue changes and help to identify specific HFpEF etiologies. Differently, the most important role of nuclear imaging [i.e., planar scintigraphy and/or single photon emission CT (SPECT)] consists in the screening and diagnosis of cardiac transthyretin amyloidosis (ATTR) in patients with HFpEF. Cardiac CT can accurately evaluate coronary artery disease both from an anatomical and functional point of view, but tissue characterization methods have also been developed. The aim of this review is to critically summarize the current uses and future perspectives of echocardiography, nuclear imaging, CT, and CMR in patients with HFpEF.

## Introduction

Heart failure with preserved ejection fraction (HFpEF) is diagnosed by identifying the HF symptoms (e.g., breathlessness, ankle swelling, and fatigue) and signs (e.g., pulmonary crackles and peripheral edema) in patients with left ventricular ejection fraction (LVEF) ≥ 50%, increased plasma natriuretic peptide (NP) levels, and specific structural alterations or diastolic dysfunction (DD) (1). Diagnosing HFpEF is essential because it represents ≥ 50% of HF cases and it is the most common form of HF in patients aged ≥ 65 years (2). Since the increased life expectancy and the increased prevalence of obesity and diabetes, it is expected to be the most prevalent form of HF in the next few years (3). Epidemiological data revealed that the prevalence of HFpEF relative to heart failure with reduced ejection fraction (HFrEF) is increasing at a rate of 1% per year. More often, the patients affected by HFpEF are women and older with risk factors and comorbidities (such as obesity, hypertension, diabetes, chronic kidney disease, and chronic obstructive pulmonary disease) (4, 5). HFpEF is associated with an adverse prognosis, similar to that of HFrEF (6). Together with clinical examination and plasma NP sampling, cardiovascular imaging plays a pivotal role in HFpEF diagnosis. However, cardiovascular imaging utility is not limited to HFpEF diagnosis only. As HFpEF is a complex syndrome that encompasses a wide range of different etiologies, cardiovascular imaging can be helpful in identifying specific HFpEF causes and therefore in tailoring specific therapies. Furthermore, data obtained from different imaging modalities can also be used to guide prognosis stratification.

## Definition And Etiology of HFpEF

Heart failure with preserved ejection fraction has been defined by the European Society of Cardiology (ESC) as a clinical entity marked by classic signs and symptoms of HF, elevated NP, and preserved LVEF (≥50%), with evidence of DD or structural heart disease (1). The diagnosis of HFpEF is challenging *per se* and this is especially true when concomitant diseases are present, for instance, atrial fibrillation (AF), where patients often present with higher NP (7). In these conditions, it is reasonable to use different N-terminal-pro brain natriuretic peptide (NT-proBNP) or BNP levels cutoff for diagnosing HFpEF according to the presence of sinus rhythm (with lower cutoffs) or AF (higher cutoffs). Furthermore, some morphologic parameters are modified in AF as compared with sinus rhythm, for instance, left atrium volume index (LAVI) is increased and functional parameters inherent DD are less well-established (8). In parallel, AF represents a worsening condition and patients with HFpEF usually have more severe HF symptoms compared with patients with HFpEF and sinus rhythm.

Actually, numerous studies have reported an intrinsic limitation of LVEF in assessing LV systolic function since it represents an imperfect marker due to various reasons (geometric assumptions, significant left ventricular hypertrophy, marked reduced longitudinal contraction with preserved LVFE, etc.). It seems that strain measurements reflect systolic function better than EF as demonstrated in numerous study populations, where a significant reduction in left ventricular global longitudinal strain (GLS) did not match with a corresponding reduction of LVEF (9). The significant reduction of GLS could be compensated by a small increase of global circumferential strain (GCS) or wall thickness or reduced diameter, so GCS may contribute more than twice as much to EF than GLS. Moreover, although reduction occurs in both longitudinal and circumferential shortening, the increased wall thickness or reduced diameter of the ventricles can maintain a preserved EF. Therefore, on the one hand, LVEF plays a key role in the management of almost any patients and its availability and reproducibility are unrivaled, and on the other hand, we know that EF alone is insufficient to identify and/or phenotype a disease (10, 11).

Heart failure with preserved ejection fraction usually represents a clinical syndrome resulting from a combination of multiple risk factors and comorbidities, comprising female sex, older, obesity, diabetes mellitus, systemic arterial hypertension, renal dysfunction, sleep disorders, chronic obstructive pulmonary disease (COPD), and anemia (2, 4, 5, 12). Compared with patients with HFrEF, patients with HFpEF have significantly higher blood pressure, lower resting heart rate, and lower levels of potassium in the plasma (13). However, it is important to underline that an older patient may present signs and symptoms typical of HFpEF along with elevated NP levels, but he/she may not be affected by HFpEF (14). It is of pivotal relevance to differentiate these two conditions and two scores, discussed later, which have been recently studied to help in diagnosing HFpEF. Notably, also aging is characterized by certain alterations in echocardiography. For instance, a decrease of LA reservoir and conduit strain are the first changes that can be found in healthy aging, followed by an impairment of LV GLS. Right ventricular (RV) strain and aging, instead, have no independent association.

The etiology and correlated pathophysiologic mechanisms underlying HF are different considering the two forms at reduced and preserved EF. It was shown that the pathophysiology of HFpEF goes far beyond DD and the essence of the pathophysiology of HFpEF is an increase of left ventricle (LV) filling pressure (15). To sum up, diastolic disfunction does not equal HFpEF. Indeed, diastolic disfunction due to aging in the absence of signs and symptoms of HF cannot be defined as a pathological condition; on the other hand, in a patient with signs and/or symptoms of HF, it is necessary to identify sufficient structural heart disease that can explain the clinical context to diagnose HFpEF.

Taken together, cardiovascular pathophysiological processes include increased systemic vascular resistance, increased conduit arterial stiffness, abnormal ventricular-arterial coupling, reduced LV long-axis systolic function, slowed early diastolic relaxation, reduced LV compliance with increased end-diastolic stiffness, reduced LA reservoir and contractile function, impaired RV function, and chronotropic incompetence (16–20). Moreover, coronary flow reserve (CFR) seems to predict the development of systolic and diastolic HF. CFR is dependent on the combined effects of epicardial coronary stenosis and microvascular dysfunction. In the absence of obstructive coronary artery stenosis, an impaired CFR reflects the presence of microvascular dysfunction. The CFR can be assessed in the left anterior descending artery by transthoracic Doppler echocardiography in a non-invasive and physiological way. So, the development of systolic and diastolic HF can be predicted by a lower CFR value with excellent sensitivity and specificity (21). It seems that the coronary microvascular abnormalities occur even in the early stage of the disease when LV contractility is preserved. Similarly, impaired coronary microcirculation and LV diastolic disfunction share the same pathogenic mechanisms [e.g., LV hypertrophy, insulin resistance, disorders of the sympathetic nervous system (SNS), etc.] and, in the end, they are linked by a close relationship.

The pathophysiological phenotype prevailing in the patient affected by HFpEF should be always established, considering that it may allow the prescription of specific therapies.

Specific etiologies underlying HFpEF-like syndromes could be classified in abnormalities of the myocardium and abnormalities of loading conditions. The first group can include ischemic disease (such as myocardial postinfarction and myocardial stunning), toxic conditions (such as recreational substance abuse, heavy metals, medications, and radiations), immune and inflammatory disease (related or not to infection, such as myocarditis and chronic inflammatory cardiomyopathy), infiltrative conditions (related or not to malignancy), metabolic pathologies (hormonal or nutritional), and genetic conditions (such as hypertrophic cardiomyopathy [HCM], restrictive cardiomyopathies, or early forms of muscular dystrophies) (22–27). The second group can include primary or secondary forms of arterial hypertension, acquired or congenital valvular and structural defects, pericardial and endomyocardial pathologies, high output states (such as severe anemia or sepsis), volume overload, and rhythm disorders (e.g., atrial/ventricular arrhythmias, pacing, and conduction disorders). They should always be considered once a diagnosis of HFpEF has been made (28, 29).

In summary, while different specific etiologies could lead to HFpEF, the most common risk factors and specific causes for HFpEF are moderate-to-severe non-controlled arterial hypertension, moderate-to-severe non-controlled diabetes mellitus, permanent AF, history or presence of severe coronary artery disease (CAD), and to a lesser extent, transthyretin amyloidosis (ATTR), respectively.

Recently, a phenotype-oriented approach to HFpEF has been proposed. Four main clinical phenotypes have been identified (aging, obesity, pulmonary hypertension, and CAD phenotype). This classification may help the management of these patients since every group implies a different therapeutical pathway (30).

## Diagnosis of HFpEF

**Figure 1:**
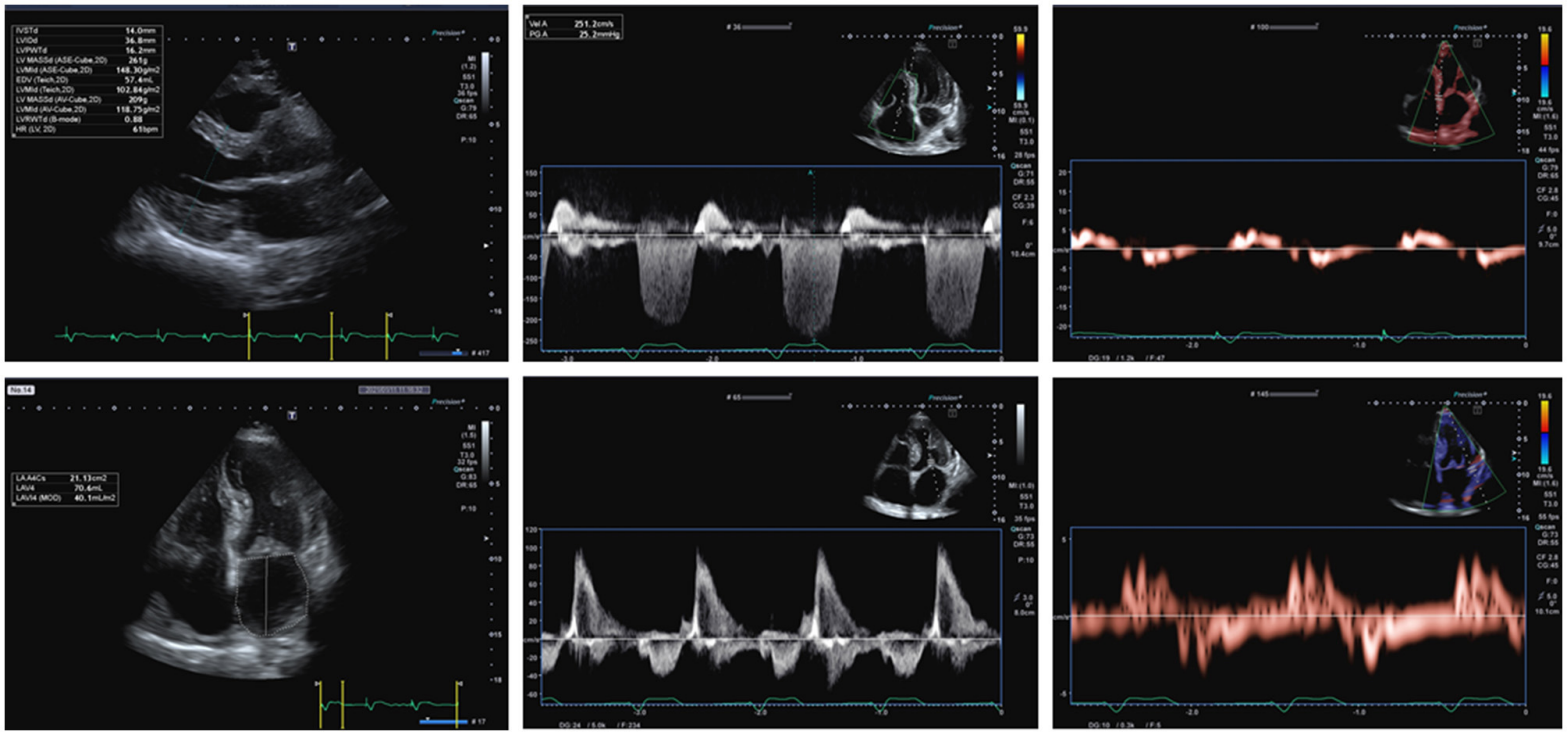
A 71-year-old male patient complained dyspnea on mild effort, fatigue, and lower limb edemas, 7 years before he was diagnosed with AL amyloidosis. More recently, he was subjected to implantation of bicameral pacemaker and after that he presented persistent atrial fibrillation. ECG showed atrial fibrillation with a ventricular paced rhythm (mean ventricular rate: 75 bpm) and low-voltage QRS complexes in limb leads. So, according to the proposed Heart Failure Association (HFA) algorithm, the pretest assessment resulted suggestive of heart failure with preserved ejection fraction (HFpEF). 2D transthoracic echocardiography (TTE) apical 4-chamber view showed significant thickening of the both ventricles (ventricular septum wall thickness = 14 mm, LV posterior wall thickness = 16 mm, right wall thickness (RWT) = 0.83, left ventricle mass indexed (LVMi) = 150 g/m^2^), biatrial enlargement [left atrium volume index (LAVI) = 40 mL/m^2^], and thickening of atrioventricular valves. 2D TTE showed preserved systolic function of left ventricle assessed by biplane Simpson's method [left ventricular ejection fraction (LVEF) 55%]. 2D TTE apical 4-chamber view with pulsed wave Doppler showed peak E velocity equal to 93 cm/s. 2D TTE tissue Doppler imaging (TDI) showed reduced septal mitral annular peak early diastolic velocity e' (3.4 cm/s) and reduced lateral mitral annular peak early diastolic velocity e' (3.6 cm/s), with a E/e' ratio equal to 26.6. Color Doppler assessment showed mild tricuspid regurgitation with TRV equal to 2.5 m/s. Blood tests showed NT-proBNP equals to 3,251 pg/mL. The Echocardiographic and Natriuretic Peptide Score (Step 2 of the proposed HFA Algorithm) is equal to 6: in the functional domain, the patient scores 2 points (major criterion: septal e' <7 cm/s, lateral e' <10 cm/s, average E/e' ≥ 15), in the morphological domain, he scores 2 points (major criterion: LVMI ≥ 149 g/m^2^ in males and RWT > 0,42; minor criterion: LAVI between 34 and 40 mL/m^2^, LV wall thickness ≥ 12 mm) and he achieves the major criterion related to NT-proBNP (>660 pg/ml in atrial fibrillation), so further 2 points can be added to the total amount. In conclusion, the diagnosis of HFpEF can be confirmed.

**Figure 2:**
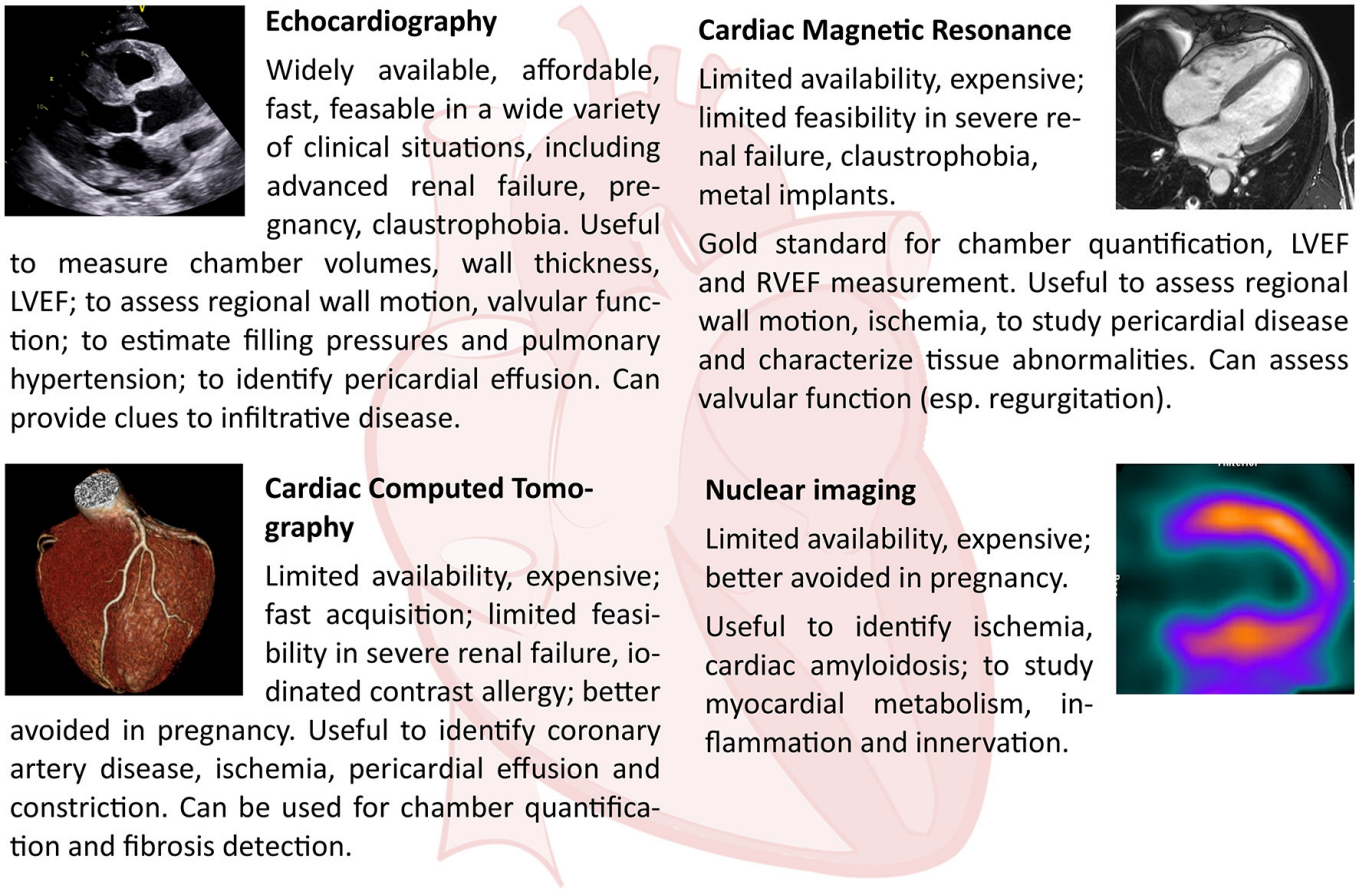
Strengths and limits of each cardiac imaging modality, and their principal applications in the diagnosis of HFpEF.

Due to the diagnostic complexity of HFpEF, some algorithms were built up in the recent past aiming to establish a probability of HFpEF diagnosis suited for each patient (31–34). In 2018, Reddy et al. developed the *H*_2_FPEF score, which is a weighted scoring system that uses six simple clinical characteristics and conventional echocardiographic information (35). One year later, a consensus recommendation from the Heart Failure Association (HFA) of the ESC was published containing a new diagnostic algorithm for HFpEF (36). This proposed score, named HFA-PEFF score, relies on four diagnostic steps and embraces recently validated functional and structural parameters together with NP assessment in a so precise fashion that confirmation or exclusion of HFpEF at the end of algorithm is highly reliable (Table 1). After initial anamnestic and clinical overview of the patient, the path of the algorithm leads to consider precise measurements by echocardiography together with NP values. Further diagnostic tools, such as cardiac magnetic resonance (CMR), cardiac CT (CCT), nuclear or other invasive diagnostics, are mainly taken in consideration in case of path clinically inconclusive (Figure 1). Figure 2 summarizes the strengths and limits of each cardiac imaging modality, and their key applications in the diagnosis of HFpEF, as detailed in the next sections.

**Table 1:** HFA-PEFF score.

**Step 1: Initial overview**		
•Comorbidities and risk factors •Symptoms and/or signs of HF •Rule out other cardiac/non cardiac causes		•Standard diagnostic tests: ECG, standard echocardiography, natriuretic peptides, ergometry, 6-MWT, Cardiopulmonary exercise testing
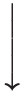		
Results suggestive of HFpEF		
**Step 2: Echocardiographic and biomarker scoring**		
**Functional domain:** *Major:* •septal e' <7 cm/s or •lateral e' <10 cm/s or •average E/e' ≥ 15 or •TR velocity > 2.8 m/s (PASP > 35 mmHg)	**Morphological domain:** *Major:*- LAVI > 34 ml/*m*^2^ in sinus rhythm or - LAVI > 40 ml/*m*^2^ in atrial fibrillation or - LVMI ≥ 149/122 g/*m*^2^ (M/W) and RWT > 0,42	**Biomarker domain:** *Major:* - NT-proBNP > 220 pg/ml or BNP > 80 pg/ml in sinus rhythm - NT-proBNP > 660 pg/ml or BNP > 240 pg/ml in atrial fibrillation
Minor: - Average E/e' 9–14 or - GLS <16%	Minor: - LAVI 29–34 ml/*m*^2^ in sinus rhythm or - LAVI 34–40 ml/*m*^2^ in atrial fibrillation or - LVMI > 115/95 g/*m*^2^ (M/W) or - RWT > 0,42 or - LV wall thickness ≥ 12 mm	Minor: - NT-proBNP 125–220 pg/ml or BNP 35–80 pg/ml in sinus rhythm - NT-proBNP 365–660 pg/ml or BNP 105–240 pg/ml in atrial fibrillation
Major and Minor criteria are scored with 2 and 1 points, respectively. Points are added only when they come from different domains.		
**≥5 points: definite diagnosis of HFpEF**		
**2–4 points: uncertain diagnosis of HFpEF**		
**Step 3: Functional testing in Case of Uncertainty**		
***Diastolic echo stress test:*** - Average E/e' ≥ 15: 2 points - Average E/e' ≥ 15 and TR velocity > 3.4 m/s: 3 points		If Echo inconclusive, perform invasive haemodynamic measurements (right heart catheterization at rest or during exercise)
**If Step (2)** **+** **Step (3)** **≥5 points ->** **definite diagnosis of HFpEF**		
**Step 4: Final etiology**		
Cardiovascular Magnetic Resonance		
Scintigraphy / CT / PET		
Cardiac or Non-Cardiac Biopsies		
Genetic testing		
Specific Laboratory Tests		

*The table shows the proposed score that relies on four diagnostic steps and embraces validated functional and structural parameters together with natriuretic peptides assessment leading the confirmation or exclusion of HFpEF*.

*HF, heart failure; ECG, electrocardiogram; 6-MWT, 6-minute walking test; HFpEF, heart failure preserved ejection fraction; TR, tricuspid valve; PASP, pulmonary artery systolic pressure; GLS, global longitudinal strain; LAVI, left atrium volume index; LVMI, left ventricle mass index; RWT, right wall thickness; NT-proBNP, Nterminal-pro Brain Natriuretic Peptide; CT, computed tomography; PET, positron emission tomography [modified from Pieske et al. (36)]*.

According to the first step of HFA-PEFF score, an initial evaluation should be performed in any patient who presents with symptoms and/or signs compatible with a diagnosis of HF [New York Heart Association functional classification (NYHA) Class II or III, orthopnea, reduced exercise capacity and fatigue, peripheral edema]. During this step, a detailed clinical and demographic history and standard diagnostic should be done. Among those, echocardiography should be used to measure LVEF (from biplane or three-dimensional images, not estimated) and LV diameters and volumes and a diagnosis of HFpEF is likely if there is a non-dilated LV with normal EF, concentric remodeling or LVH, and LA enlargement. Blood tests should include NP measurement, and elevated levels suggest heart disease despite normal levels do not exclude diagnosis of HFpEF. A standard exercise stress test can be included in the etiological workup to identify myocardial ischemia, abnormal blood pressure response to exercise, chronotropic incompetence, or supraventricular and ventricular arrhythmias.

Once step 1 is positive, the usefulness of step 2 consists in confirmation or exclusion of HFpEF diagnosis and this is why highly detailed echocardiographic measurements are warranted. The complexity of the algorithm is due to the limited accuracy of each echocardiographic parameter so that a combination of functional and morphological measurements and biomarker levels is able to profile a correct diagnostic classification. Importantly, each diagnostic cutoff depends on specific variables, such as age, gender, body weight, and renal function, and in case of AF, this variability is so relevant that different values need to be explicated and weighted. According to the severity of an abnormality, it is recommended as the distinction between major and minor diagnostic criteria, characterized by a higher specificity and sensitivity, respectively. Finally, the only biomarker considered in HFA-PEFF score is represented by NT-proBNP, which has a different weight in the score according to the cutoff values in sinus rhythm and AF. Definitive cutoffs to diagnose HFpEF in patients with synus rhythm (SR) or in AF are not well-established, and trials have used different values (37, 38). In the setting of screening, average NPs have been reported to be 3–3.5 fold higher in patients with AF than in patients with SR (8, 39).

In a sequential logic, an uncertain diagnostic definition of HFpEF after a morphological evaluation leads to functional analysis aiming to elucidate the presence or not of HF after the applications of stress tests. Exercise echocardiography represents the first choice followed by invasive hemodynamic approach (right heart catheterization at rest or during exercise) when inconclusive or not feasible. If hemodynamic abnormalities such as reduced stroke volume, reduced cardiac output (CO), and elevated LV filling pressures are detected either at rest [left ventricular end-diastolic pressure ≥ 16 mmHg, pulmonary capillary wedge pressure (PCWP) ≥ 15 mmHg at rest] or during exercise, it is possible to confirm that the symptoms complained by the patients originate from the heart (32, 40, 41). The last step of HFA-ESC algorithm is designed to identify a specific etiology, if appropriate, once HFpEF has been diagnosed. Indeed, the possibility to recognize a specific underlying etiology has to be considered for therapeutic purposes. This goal may be achieved by different diagnostic approach, such as CMR, CCT, or nuclear techniques, depending on specific cases, other than local availability and expertise.

The HFA-PEFF algorithm and *H*_2_FPEF score are surely useful tool in the diagnosis of HFpEF, since the high-likelihood cutoff of either score are quite accurate to diagnose this syndrome. However, the diagnostic uncertainty in HFpEF implies that some patients may be differently classified depending on which score we apply. AF and body mass index (BMI) seem to represent the main players of the discrepancy between the scores (42).

Since the diagnostic performance of both *H*_2_FPEF score and HFA-PEFF algorithm has varied and the additional tests recommended in case of uncertainty are not widely available except in specialized centers, none of the abovementioned scores have been recommended in the 2021 ESC HF guidelines, which contain the latest recommendations on this topic (1, 43, 44). So, the current guidelines recommend a simplified approach, instead of using a diagnostic score, leaving the HFA-PEFF diagnostic algorithm to those who have access to expertise. Cardiopulmonary exercise testing, exercise stress testing, and invasive hemodynamic testing still represent additional confirmatory evaluation (Table 2).

**Table 2:** Proposed scores for diagnosis of HFpEF.

	**Diagnostic criteria**	**Comment**
H2FPEF score	H, Heavy: BMI > 30 kg/m^2^ (2 points) H, Hypertension: use of ≥2 antihypertensive drugs (1 point) F, Atrial Fibrillation (3 points) P, Pulmonary hypertension: PAPS > 35 mmHg (1 point). E, Elderly: age > 60 years (1 point). F, Filling: elevated filling pressure, E/e > 9 (1 point).	The probability of HFpEF increases with H2FPEF score: - 0–1: low probability (<20%), unlikely HFpEF. - 2–5: intermediate probability, need of additional testing (echocardiographic or invasive hemodynamic exercise stress tests). - 6–9: high probability (>90%), HFpEF is likely.
HFA-PEFF algorithm	Step 1: Initial overview Step 2: - Echocardiographic and Biomarker Scoring - Functional domain - Morphological domain - Biomarker domain Step 3: Functional testing in case of uncertainty Step 4: Final etiology	***Step 1*** is useful to identify patient with clinical suspicion of HFpEF and to exclude other causes. ***Step 2*** requires a detailed echocardiography + assessment of NPs levels. The measurements are grouped in domain and classified in major and minor criteria; if they are fulfilled, they add 2 points or 1 point respectively. Total score: ≥ 5 points is diagnostic of HFpEF 2–4 points requires further evaluation (Step 3) ≤ 1 HFpEF diagnosis very doubtful. ***Step 3*** diastolic echo stress test and invasive measurements (when required). Step 2 score plus Step 3 score ≥ 5 points, the diagnosis of HFpEF is confirmed. ***Step 4*** encourages the research of a specific etiology of HFpEF since it can affect the therapeutic decision.
2021 ESC guidelines	All the following criteria need to be fulfilled: - Symptoms and/or signs - LVEF ≥ 50% - Objective evidence of cardiac structural and/or functional abnormalities consistent with the presence of LV diastolic dysfunction/raised LV filling pressure.	The recommended parameters are the following: - LV mass index (≥95 g/m^2^ for female, ≥115 g/m^2^ for male); - Relative wall thickness (>0.42); - LA volume index (>34 mL/m^2^ for patients in sinus rhythm, >40 mL/m^2^ for patients in atrial fibrillation); - E/e' ratio at rest (>9); - NPs blood concentration (NT-proBNP >125 pg/mL or BNP >35 pg/mL in case of sinus rhythm; NT-proBNP >365 pg/mL or BNP >105 pg/mL in case of atrial fibrillation); - PA systolic pressure (>35 mmHg); - TR velocity at rest (>2.8 m/s). The probability of HFpEF proportionally increases with the number of the parameters.

*BMI, body mass index; PASP, pulmonary artery systolic pressure; HFpEF, heart failure preserved ejection fraction; LVEF, left ventricle ejection fraction; NT-proBNP, Nterminal-pro Brain Natriuretic Peptide; PA, pulmonary artery; TR, tricuspid valve*.

Recently, the European Association of Cardiovascular Imaging (EACVI) has published an expert consensus document about the importance of multimodality imaging in patients affected by HFpEF, since it can be very helpful to determine specific etiologies (45, 46). CAD, HCM, cardiac amyloidosis, Fabry disease, and sarcoidosis are the main identified etiologies of patients with HFpEF, and it is important to highlight in these setting, the possibility of prescribing established and specific therapies. Echocardiography plays a central role, and it can be sufficient for a conclusive diagnosis in case of cardiomyopathy with specific findings (e.g., HCM). Differently, some patients need confirmation by additional imaging, such as CMR, coronary angiography (CT or invasive), positron emission tomography (PET), single photon emission CT (SPECT), bone and cardiac scintigraphy, right heart catheterization at rest, or during exercise. In a limited number of patients, a myocardial biopsy may be helpful. The consensus document recommends to rule out alternative diagnoses, like non-cardiac types of pulmonary hypertension or constrictive pericarditis. For instance, it should be taken into consideration in the differential diagnosis of suspected HFpEF other causes of dyspnea such as pulmonary embolism, severe renal failure, pneumonia, and decompensated COPD.

## Echocardiography

### Diastolic Function and HFpEF

In 1997, Nishimura et al. published a cornerstone paper about the assessment of diastolic filling of the left ventricle using Doppler echocardiography, in particular, mitral flow velocity curves (47), which has been updated about 10 years later by a key publication of Lester et al. (48).

More recently, in 2016, an update from the American Society of Echocardiography (ASE) and EACVI about the recommendation for the evaluation of left ventricular diastolic function by echocardiography was published (15). It established how to determine elevated left ventricle filling pressures (LVFP) in patients with signs and symptoms of HF and with the myocardial disease using echocardiography. Mitral flow velocities, mitral annular e′ velocity, E/e′ ratio, peak velocity of tricuspid valve regurgitation (TR) jet, and LA maximum volume index are the main values recommended for the assessment of LV diastolic function grade. Additional variables are pulmonary vein velocities and LV, GLS measured by speckle-tracking echocardiography (STE), the last one used to identify mild reduction in systolic function.

Initially, it is necessary to rule out the presence of AF, significant mitral valve disease (at least moderate mitral annular calcification, any mitral stenosis or mitral regurgitation of more than moderate severity, mitral valve repair or prosthetic mitral valve), LV assist devices, left bundle branch block, and ventricular paced rhythm because of the inaccuracy of the mitral E/e′ ratio in this setting.

The recommended approach is mainly based on the mitral E/A ratio and it is indicated for patients in sinus rhythm. Patients with E/A ratio ≥ 2 are considered as having elevated LA mean pressure and grade III DD, thereby the diagnosis of HFpEF could be established. In patients treated with recent cardioversion to sinus rhythm, mitral deceleration time (DT) should be added to the assessment of LV diastolic function because they can have a markedly reduced mitral A velocity for the LA stunning at the time of the echocardiographic examination; this can lead to an E/A ratio ≥ 2 despite the absence of elevated LV filling pressures. Moreover, an E/A ratio >2 can be a normal finding in young individuals (<40 years of age) (49) and therefore in this age group, other signs of DD should be looked for.

Notably, in patients with mitral E/A ratio between 0.8 and 1.9 or with mitral E/A ratio ≤ 0.8 and peak E velocity > 50 cm/s, further three criteria should be considered for finally determining elevated LV filling pressures (50–52): left atrial volume index (LAVI) > 34 mL/m^2^; peak velocity of tricuspid regurgitation (TR) by CW Doppler obtained from multiple views > 2.8 m/s; and mitral average septal-lateral E/e′ ratio > 14. All three indices have been shown to be of values in identifying patients with HFpEF. The pulmonary venous S/D ratio is often <1 in healthy young individuals, so this index has a limited value in patients with normal LVEF. If all three parameters are available and only one of the three or none of the three meets the cutoff value, then left atrial pressure (LAP) is normal and there is grade I DD. In addition, in patients with two of these parameters negative, further evaluation (e.g., a diastolic stress test) should be considered to confirm the diagnosis of HFpEF.

The left ventricle DD causes LA enlargement, which can lead to AF (53–55). In patients with AF, Doppler assessment of LV diastolic function is limited by the variability in cycle length, the absence of organized atrial activity, and the frequent occurrence of LA enlargement regardless of filling pressures (56). Other Doppler measurements that can be applied include peak acceleration rate of mitral E velocity (≥1.90 cm/s^2^), isovolumic relaxation time (IVRT) ( ≤ 65 ms), DT of pulmonary venous diastolic velocity ( ≤ 220 ms), E/mitral Vp (E/Vp; ≥1.4), and E/e′ ratio (≥11) (55, 57, 58). At present, the two most important criteria to determine elevated LV filling pressures in patients with AF are septal E/e' ≥ 11 and/or TR > 2.8 m/s (15, 46).

Importantly, the HFA-PEFF algorithm and the 2021 ESC HF guidelines do not add other assessment to the diagnostic pathway in case of AF and those presented two different thresholds depending on the presence of the arrhythmia: LAVI should be >40 mL/m^2^ to define LA enlargement and NPs levels should be higher to reveal raised LV filling pressures (NT-proBNP should be >365 pg/ml and BNP >105 pg/ml).

### Exercise Diastolic Stress Echocardiography

Echo stress technique may be employed in cardiology for multiple purposes including the evaluation of myocardial viability, inducible ischemia, sisto/diastolic HF, and assessment of therapeutic options (59, 60). In some patients with HFpEF, who have symptoms such as dyspnea only during exercise, often echocardiography at rest can be normal (1, 15, 36). In this regard, several studies demonstrated that in some patients with HFpEF, LV diastolic abnormalities occur only during exercise. So, adding diastolic analysis during exercise can increase the sensibility to diagnose HFpEF (41, 60, 61). Consequently, in case of suspicion of HFpEF but inconclusive criteria by using diastolic measurements at rest, a diastolic stress test should be done (1, 31, 36). Indeed, the failure of earlyvs diastolic relaxation together with increment of LV filling pressure let simpler the diagnosis of HFpEF. The parameters that have been studied most often, during or immediately after exercise, are the mitral E/e' ratio and the TR peak velocity, which indicate increases in mPCWP and pulmonary artery systolic pressure (PAPs), respectively (15, 62–64). Inconclusive results are considered when mitral E/e′ septal-lateral ratio >14 (or mitral E/e′ septal ratio >15) and at the same time TR velocity ≤ 2.8 m/s are present (65–67). Indeed, a positive stress test is considered when average E/e' > 14 (or septal E/e' > 15) and TR > 2.8 m/s are present.

The most validated and recommended protocol for a diastolic stress test is a semi-supine bicycle test with imaging during exercise or a treadmill or upright bicycle exercise protocol with imaging at or immediately after peak stress (15, 68), but there are no universally adopted protocols.

### Left Atrial Dimensions and Dysfunction

The left atrial dimensions and function should be accurately evaluated when assessing diastolic function in patients with preserved EF. Importantly, the enlargement of LA is strongly suggestive of chronically elevated LV filling pressure once pathological conditions such as atrial tachyarrhythmias and hemodynamically severe valve diseases have been excluded. The increased LV filling pressure causes LA remodeling and disfunction, which lead to worsen symptoms, pulmonary vascular disease, greater RV dysfunction, depressed exercise capacity, and adverse outcomes (69–72).

The left atrium pathophysiology and its mechanics generally talking have a pivotal role in diagnosis and prognosis of HFpEF, and indices of LA mechanics have utility in HFpEF (73, 74). A study published by Morris et al. showed that abnormal LA strain (<23%) is significantly associated with worse NYHA class and with the risk of HF hospitalization at 2 years independently from age and sex (73). Very recently, a meta-analysis correlated LA disfunction parameters with outcomes in patients with HFpEF showing that decreased LA reservoir strain independently predicted for all-cause mortality or HF hospitalization (75). In conclusion, left atrial size is nowadays the best marker of chronic elevation of filling pressures, but the assessment of left atrial function, as discussed above, should be addressed in future clinical research in order to be included in future guidelines.

### Left Ventricle Systolic Dysfunction

An impaired LV longitudinal systolic function and impaired ventricular contractility have been found in patients affected by HFpEF and can cause the symptoms, along with DD (76, 77). Importantly, LV global longitudinal strain has shown that the longitudinal systolic function of the LV seems to be significantly altered in a high proportion of patients with HFpEF. Moreover, impaired LV systolic mechanics in HFpEF also predict an increased risk of adverse outcomes (69, 78, 79).

## Cardiac Magnetic Resonance

### Morphology and Systolic and Diastolic Function Assessment

Cardiac magnetic resonance offers superior anatomical assessment, can reliably depict various diastolic events, and provides unprecedented *in vivo* tissue characterization to help the clinician to identify specific etiologies (Table 3).

**Table 3:** CMR sequences and their utility in the assessment of cardiac chambers anatomy, left ventricular diastole, and myocardial tissue in patients with HFpEF.

	**CMR sequences**	**Applications**
Morphological assessment	bSSFP	Accurately measuring LV volumes, wall thickness, mass and LVEF, without geometric assumptions
	bSSFP	Accurately measuring RV volumes, wall thickness and RVEF, without geometric assumptions
	bSSFP	Accurately measuring LA volumes and LAEF, without geometric assumptions
Functional diastolic evaluation	bSSFP	Measuring LV volume-time curve, peak filling rate, time to peak filling
	Phase-contrast, 4D-flow	Measuring mitral diastolic flow, pulmonary vein flow
	Myocardial tagging	Measuring LV recoil rate and circumferential-longitudinal shear
	bSSFP, feature tracking in post-processing	Measuring LV diastolic longitudinal, circumferential, and radial strain and strain rate
	Tissue phase-contrast	Measuring early diastolic mitral septal velocity
	CMR elastography	Measuring LV stiffness
Tissue characterization	LGE	Detecting necrotic myocardium, fibrosis
	T1 mapping (e.g., MOLLI, shMOLLI, SASHA)	Altered in fibrosis, myocardial edema, iron overload, intracellular deposition
	T2 mapping	Detecting myocardial edema
	ECV mapping	Detecting fibrosis, extracellular matrix alterations (e.g., amyloid deposition)

Cardiac magnetic resonance is the gold standard for measuring biventricular volumes, wall thickness, mass, and EF (80, 81). Modern balanced steady-state free precession (bSSFP) sequences generate images with high spatial, temporal, and contrast resolution. Left ventricular (LV) mass and volumes are measured from a set of contiguous short-axis cine slices; volumes are obtained without geometric assumptions with the summation disk method, multiplying endomyocardial areas and interslice length, and mass is calculated taking into account the myocardial specific gravity of 1.05 g/ml. Left ventricular geometric indices do not appear to have prognostic value in patients with HFpEF. Indeed, CMR-derived LV mass has been shown to predict incident HF events in a large general cohort from the Multiethnic Study of Atherosclerosis (MESA) study but not in patients with HFpEF (82, 83).

Cardiac magnetic resonance can also assess LV diastolic function by different approaches. By measurement of LV volume in different cardiac cycle phases, it is possible to derive left ventricular volume-time curves, their derivative dV/dt with peak filling rate and time to peak filling. These indices describe the speed of LV relaxation in early diastole (84). Due to lengthy manual endomyocardial contour tracing, this analysis is usually restricted to research purposes. However, artificial intelligence–assisted methods for endomyocardial border detection could accelerate analysis and make it more widely adopted in clinical practice (85). Phase-contrast technique allows to accurately measure blood flows that are perpendicular to properly placed imaging planes, and it can be used to measure CMR-equivalents of echocardiography diastolic indices such as mitral E/A ratio and pulmonary vein flow. In this regard, previously published comparative studies between the two techniques have shown good correlation (86, 87). Phase-contrast sequences are usually acquired during multiple cardiac cycles with retrospective cardiac gating. The accuracy of phase-contrast-derived measures can be worsened by arrhythmias and patient movements and is strictly dependent on the correct imaging plane prescription, which is made particularly challenging by the continuously changing mitral annulus position during the cardiac cycle. This limitation has been overcome by novel 4D-flow sequences that produce three-dimensional velocity encoded datasets, so that postprocessing multiplanar navigation allows to measure flows across any desired plane (88). Three-dimension velocity encoded imaging with retrospective mitral valve tracking results in superior accuracy than standard phase-contrast sequences when compared to echocardiography (89). Another CMR technique to evaluate diastolic function is myocardial tagging, which enables to study local myocardial deformation: specific radiofrequency impulses are applied prior to the imaging sequences to create a grid of low-intensity lines; as the heart contracts during systole and relaxes during diastole the grid is deformed. Recoil rate and circumferential-longitudinal shear indices describe early diastolic LV untwisting during isovolumic relaxation and are associated with LV pressure fall and relaxation time constant τ (90). As the tag grid rapidly fades, diastolic evaluation by myocardial tagging is limited to the early diastolic phase. Moreover, myocardial tagging is routinely used for qualitative, inspective analysis, while time-consuming quantitative methods are usually reserved for research purposes. Such limitations have been overcome by feature tracking technique, which enables to measure systolic and diastolic myocardial strain and strain rate, by tracking anatomical features in LV myocardium along the cardiac cycle, in a fashion similar to echocardiography speckle tracking (91). Feature tracking analysis is performed on common bSSFP cine images and does not require additional sequences and scan time. Feature tracking–derived LV early diastolic global longitudinal, circumferential, and radial strain rates were different in a group of 84 hypertensive patients with HFpEF compared with healthy controls and hypertensive patients, and were associated with symptoms and NP levels (92). Left ventricular early diastolic circumferential strain rate has also been shown to be reduced in older obese HFpEF patients, but has poor correlation with peak oxygen consumption (93). Another technique to depict regional myocardial deformation is tissue phase-contrast analysis, which is conceptually similar to an echocardiography tissue Doppler study. Tissue phase-contrast analysis allows to measure early diastolic mitral septal velocity (Ea); this measure, coupled with mitral valve inflow phase-contrast analysis, enables to derive septal E/Ea ratio. In line with echocardiography, CMR-derived E/Ea <8 has 100% positive predictive value for PCWP ≤ 15 mmHg, and E/Ea ratio >15 has 100% positive predictive value for PCWP > 15 mmHg (94). Using mitral inflow and myocardial tissue phase-contrast analysis, the CMR can classify DD with excellent agreement with echocardiography (95). The tissue phase-contrast analysis can also measure different components of wall motion (radial, longitudinal, and circumferential) during both systole and diastole, using a respiratory self-navigated, cardiac-gated, velocity-encoded golden-angle spiral sequence (96). All of the above-mentioned techniques do not allow to measure LV stiffness, but its effects and surrogate markers. A promising approach enables to directly measure LV stiffness with a 3D high-frequency CMR elastography technique, also generating LV stiffness maps (97).

Cardiac magnetic resonance can also assess left atrial (LA) area and volume, using the biplane area-length method from the 4-chamber and 2-chamber planes, or using the disc summation method from a cine short-axis stack encompassing the LA. Due to its different roles during the cardiac cycle (reservoir, conduit, pump), the LA function can be investigated by different indices and techniques, from volumetric to feature-tracking methods. Left atrium EF is calculated dividing the difference between maximum and minimum LA volumes by maximum LA volume. It is associated with increased LV end-diastolic pressure and is a strong and independent prognostic predictor in HFpEF (98, 99).

Finally, CMR is the gold standard for measuring RV and atrial volumes and function (80). RV systolic dysfunction is highly prevalent in HFpEF, and is associated with worse symptoms and prognosis (100, 101).

### Tissue Characterization

Due to its high superior contrast resolution and intrinsic multiparameter nature, CMR can accurately characterize myocardial tissue conditions such as edema, fibrosis, and infiltrative processes. Kanagala et al. showed that CMR with tissue characterization could identify previously undiagnosed pathology in 27% of 154 patients with HFpEF; notably diagnoses made by CMR conferred an increased risk of adverse outcome (102).

Late gadolinium enhancement (LGE) imaging technique takes advantage of different wash-out kinetics of gadolinium-based contrast from normal myocardium and necrotic myocardium or fibrosis (103). LGE enables to differentiate viable from non-viable myocardium, ischemic from non-ischemic cardiomyopathies, and plays a pivotal role in diagnosing different non-ischemic cardiomyopathies (104–108). Moreover, LGE presence and extent have been reported to hold prognostic value in different cardiomyopathies (109–112). In 111 patients with HFpEF, LGE extent was an independent predictor of cardiovascular death or decompensated HF admission, even after adjustment for age, diabetes mellitus, functional class, LVEF, and history of HF hospitalizations (113).

**Figure 3:**
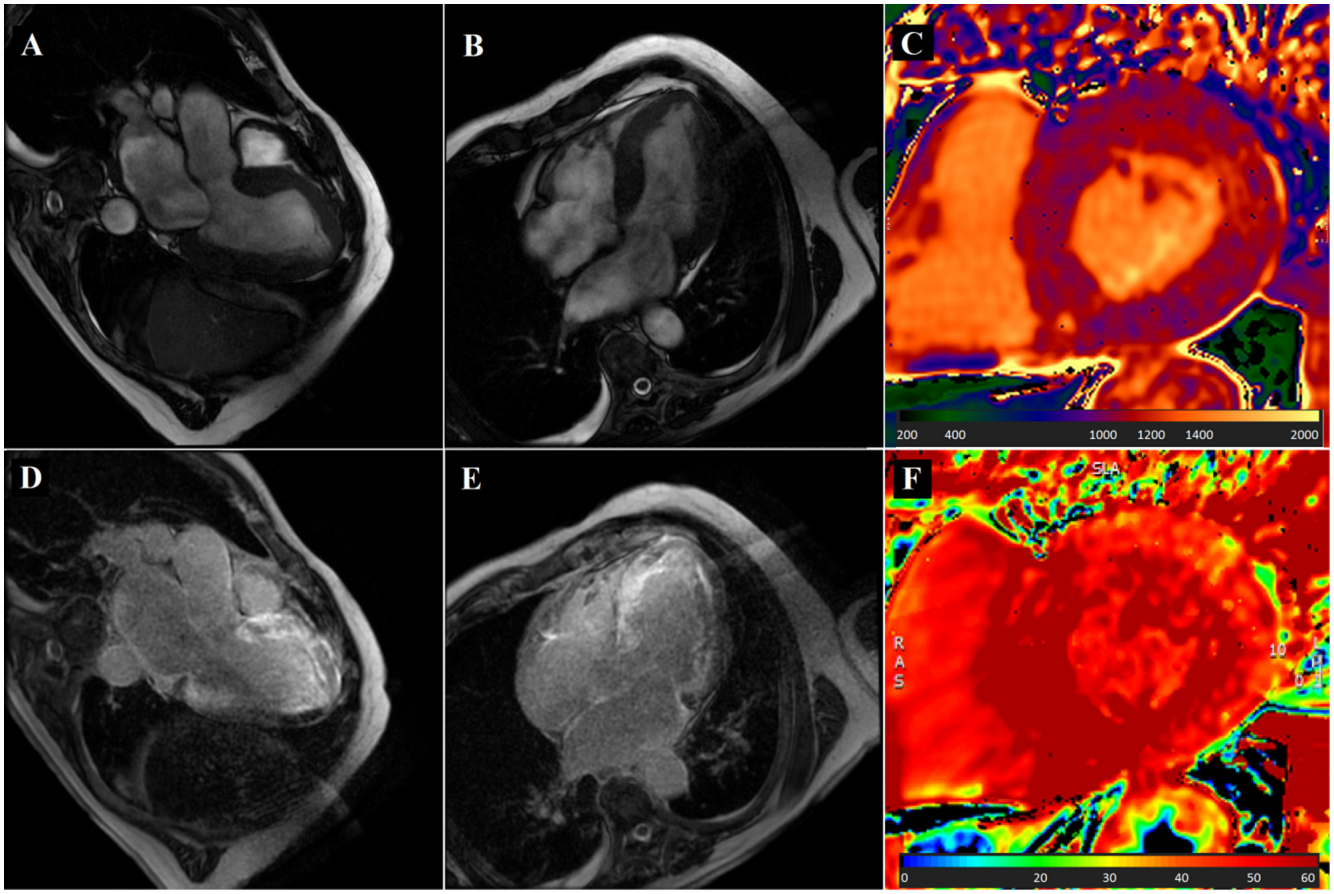
Cardiovascular magnetic resonance (CMR) in a patient with dyspnea and cardiac amyloidosis. An 80-year-old man with a history of ischemic heart disease was referred to the cardiology clinic because of dyspnea. Clinical examination was unremarkable. CMR showed increased left ventricular wall thickness [**(A,B)**, bSSFP images showing three- and four-chamber view respectively], increased left ventricular mass (115.9 g/m^2^), and small pleural and pericardial effusion. Native T1 values were significantly increased, up to 1,200 ms **(C)**. Late gadolinium enhancement showed diffuse left ventricular subendocardial enhancement, also involving the atria [**(D,E)**, three- and four-chamber view, respectively]. ECV was markedly increased **(F)**. Patient was diagnosed with cardiac amyloidosis.

Quantitative methods for native longitudinal relaxation time (T1), transverse relaxation time (T2), and extracellular volume (ECV) mapping have been developed to identify both focal and global myocardial changes. In particular, native T1 mapping, which is obtained without the administration of gadolinium contrast agent, may reveal diffuse myocardial fibrosis and can identify subtle changes that would go unnoticed by LGE. Differently, ECV can be calculated from native and postcontrast T1 values and shows high agreement with extracellular space as a whole (including collagen, extracellular matrix proteins, and vessels) (114). Native T1 mapping can help in identifying acute and chronic myocardial infarction, myocarditis, amyloidosis, Fabry disease, and iron overload (115–120) (Figure 3). Native T2 mapping can detect myocardial edema in acute myocardial ischemia and inflammatory cardiomyopathies (121, 122). Fibrosis and extracellular matrix alterations are thought to be the major contributors to DD and HFpEF. ECV is higher in patients with HFpEF than in controls and is associated with DD (123). Moreover, higher ECV is associated with higher peak filling rate, a CMR-derived left ventricular DD index, higher invasively measured left ventricular stiffness, prolonged active left ventricular relaxation, LV mass, and maximal left atrial volume (124, 125). Higher ECV is also associated with higher NP levels and worse functional class and outcome. In 250 patients who were at risk of HFpEF, given their elevated NP levels, higher ECV was associated with worse outcome, and the authors hypothesize that myocardial fibrosis as detected by ECV might precede over HFpEF diagnosis (126). Notably, in a study on 19 patients with HFpEF, global native T1 was associated with increased ECV, invasive LV stiffness, and histological fibrosis (127). Native T1 mapping has been proposed as a method to investigate patients with HFpEF with severely reduced renal function, who cannot be administered gadolinium-based contrast agents. Moreover, anterior RV insertion point native T1 values are thought to reflect increased RV afterload and hold prognostic value in HFpEF (128).

Right ventricular free wall ECV can be obtained using high-resolution mapping sequences. In 14 patients with HFpEF and pulmonary hypertension, RV free wall ECV was associated with higher RV end-diastolic volume and worse RV free wall strain, independently of invasively measured pulmonary resistance (129).

## Nuclear Imaging

Traditionally, the evaluation and management of the patients affected by HF have been focused on hemodynamic abnormalities, but the neurohormonal and molecular pathophysiologies have gained attention along with the rapid development of nuclear medicine instruments and the widespread availability of new radiopharmaceutical agents. The metabolic assessment, consisting in identifying ischemic but viable myocardium, can be done; thanks to PET. This is useful in order to detect CAD and, basically, as a first step to determine HF etiology.

While fluorodeoxyglucose (FDG)-positron emission tomography (PET) is available only in limited number of centers, SPECT using ultrahigh energy collimator and branched fatty acid analog I-123 beta-methyl-p-iodophenylpentadecanoic acid (BMIPP) represents a solid alternative for metabolic imaging in routine clinical settings (130). A reduced BMIPP uptake at rest, despite normal perfusion, identifies severe ischemia, since BMIPP plays a central role in ischemic memory imaging. Moreover, quantitative blood flow techniques, such as CFR, seem to be interesting to overcome potential underestimation of ischemia. CFR can be assessed by cardiac PET with either rubidium-82 (82Rb) or nitrogen-13 ammonia (13NH3) as radiotracer (131).

In the pathophysiology of HF, the activation of the SNS plays a key role (132, 133). Indeed, the SNS hyperactivity and the resultant compromised myocardial sympathetic innervation have been demonstrated to contribute to the DD and to predict adverse cardiac events in patients with HF (134, 135).

While the sympathetic innervation in HFrEF has been studied by several authors, the same cannot be said for HFpEF. It has been found that in patients with HFrEF, excessive cardiac SNS activation is typical of HF progression. An increase in adrenergic drive and consequent downregulated uptake-1 mechanism generates desensitization/downregulation of b-adrenergic receptors, cardiac remodeling, and thus progression of HF (132, 136, 137). Grassi et al. showed that SNS hyperactivity can cause DD in hypertensive patients. Actually, patients presenting DD and affected by hypertension show higher SNS activity [e.g., muscle sympathetic nerve activity (MSNA)] and abnormal baroreflex modulation than patients affected by hypertension without DD, and both these groups have higher MSNA than age-matched controls (138). Notably, planar 123I-metaiodobenzylguanadine (123I-mIBG) scintigraphy, which is able to determine the sympathetic presynaptic nerve function, is applicable in HFpEF for the correlation with the severity of DD, LV remodeling, functional capability of the patient, and his/her response to therapy (139–141).

In 2017, Aikawa et al. studied the relationship between impaired cardiac SNS innervation and LV DD (assessed by transthoracic echocardiography) in patients with HFpEF using PET imaging with 11C-hydroxyephedrine (11C-HED) and showed that 11C-HED uptake was globally reduced and more heterogenous than in age-matched controls (142). Moreover, in patients with more severe DD (grade 2-3), the reduction in global 11C-HED uptake was greater with more heterogenous uptake than in patients with less severe DD (grade 0–1). While the relationships with 123I-mIBG planar scintigraphic indices in patients with HFpEF were already studied, the use of 11C-HED PET imaging represents a turning point because it allows an improved global and regional quantification of SNS innervation; thanks to better spatiotemporal resolution and more quantitative image analysis than conventional 123I-mIBG planar imaging.

It is important to underline that the most relevant role of nuclear medicine (i.e., planar scintigraphy and/or SPECT) is in the screening and diagnosis of cardiac ATTR amyloidosis in patients with HFpEF.

## Cardiac CT Angiography

**Figure 4:**
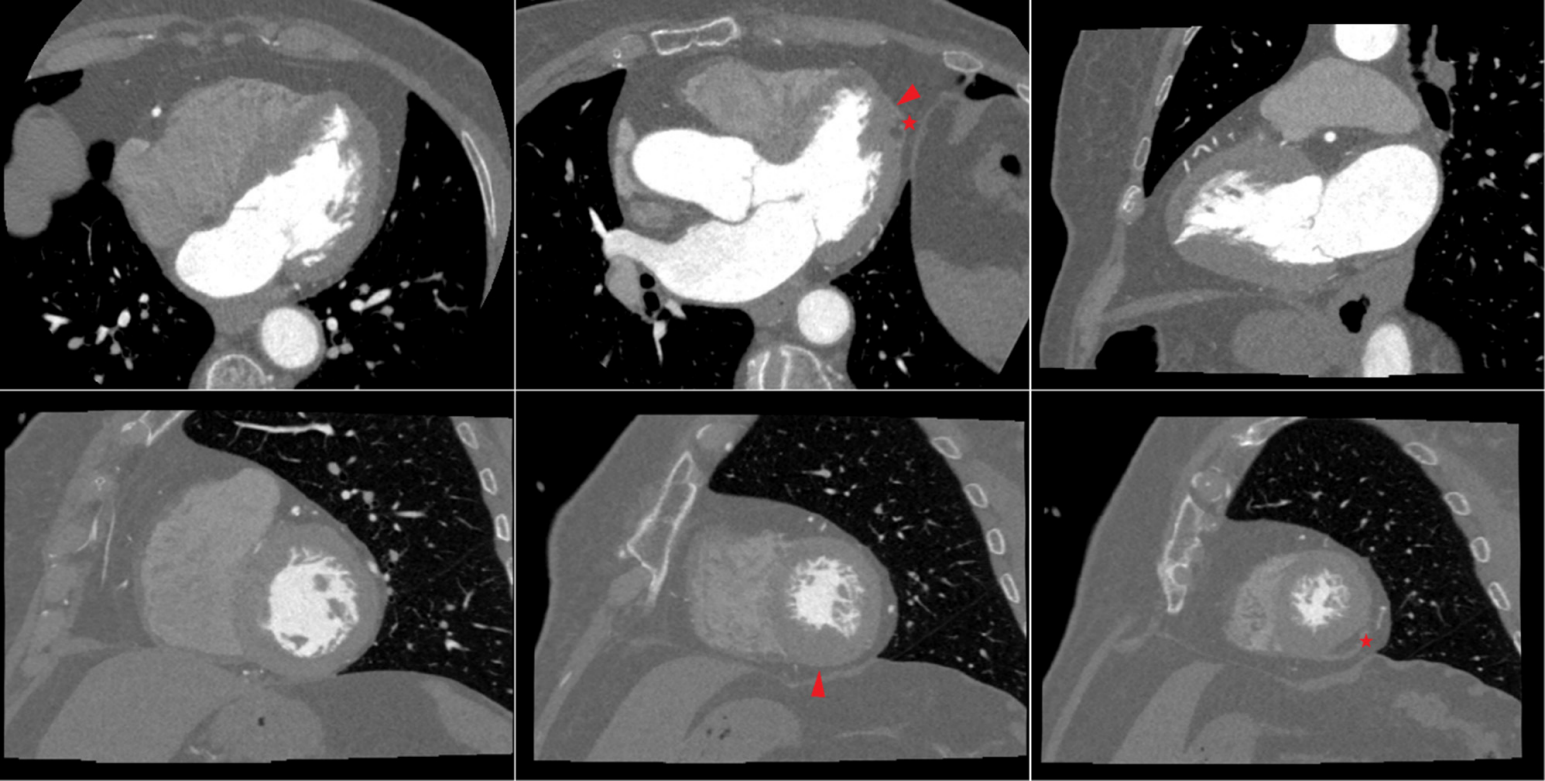
Cardiac CT in a patient with dyspnea and apical hypertrophic cardiomyopathy (left ventricular long-axis views, top row; left ventricular short axis views, bottom row). A 79-year-old woman with hypertension was referred to the cardiology clinic because of dyspnea. Clinical examination was unremarkable. Resting ECG showed anterior T wave inversion. A cardiac CT ruled out obstructive coronary artery disease, while showing hypertrophy (maximal wall thickness 15 mm at end-diastole) of the inferior and lateral apical segments, and of the inferior mid-ventricular segment (arrowhead). Moreover, there was fatty infiltration in the apical lateral segment (asterisk). Patient was diagnosed with apical hypertrophic cardiomyopathy.

During the last decades, cardiac CT angiography (CCTA) showed a dramatic technological improvement, absolutely more than any other cardiac diagnostic technique, with consequent clinical implications both in ischemic heart disease and in other cardiac context (143–148) (Figure 4). In patients presenting with dyspnea and HFpEF, CCTA can accurately rule out CAD, particularly when pretest probability is low to intermediate (149). However, CCTA also enables to accurately measure ventricles volumes, permits to assess LV function, and allows myocardial tissue characterization (150). CCTA permits multiplanar reconstruction and can evaluate cardiac structures that are typically difficult to assess with echocardiography, such as left ventricular apex, the anterolateral wall, and the atria (151). It is possible to assess left ventricular function and wall motion acquiring a whole cardiac cycle using ECG-gated scanning (152, 153), at the expense of increased radiation exposure. Iodinated contrasts have similar wash-out kinetics from normal and necrotic/fibrotic myocardium as gadolinium-based contrasts. Therefore, delayed contrast-enhanced CCTA is able to identify acute and chronic myocardial infarction and can be used to differentiate between ischemic and non-ischemic causes of left ventricular systolic dysfunction (154). In a fashion similar to CMR, ECV can be calculated from blood pool and myocardium radiodensity before and after contrast injection (155, 156). Drawbacks of delayed contrast-enhanced CCTA are prolonged scan time and increased patient radiation exposure. As far as we know, tissue characterization CT capabilities have not yet been studied in patients with HFpEF.

Valvular calcifications can be easily identified by cardiac CT; valvular calcification progression is strongly associated with HFpEF incidence (157).

Finally, cardiac CT also allows to accurately assess the presence and the extension of epicardial adipose tissue. Epicardial adipose tissue has been linked to hemodynamic abnormalities such as higher cardiac filling pressures and greater pericardial restraint, and to poorer exercise capacity in patients with the obese phenotype of HFpEF (158).

## Final Considerations on Specific Patient Populations

### End-Stage Kidney Disease (or Patients With Dialysis)

Patients with end-stage kidney disease (ESKD) often present volume overload, even without structural or functional heart disease. In addition, the symptoms typical of HF can be intermittent in patients with dialysis. Therefore, it is crucial to perform echocardiography in this subgroup of patients in order to assess systolic and diastolic ventricular function, chamber volumes, wall thickness, valve function, and filling pressures (159). Even though the role of NPs is unclear in case of dialysis, their relative negative predictive value is significant and the presence of high plasma levels in this setting of patients can be caused by both worsening of eGFR and cardiac dysfunction (160–163).

Moreover, Otsuka et al. studied serum sensitive cTroponin I (cTnI) levels in patients with ESKD. In patients with preserved LVEF underwent to dialysis, diastolic disfunction, and risk of mortality were significantly associated with sensitive cTnI levels regardless of echocardiographic variables (164). Patients with elevated cTnI level showed a greater E/e' ratio and LV mass index, assessed by echocardiography, which can be the underlying mechanism of troponin elevation in patients with dialysis and preserved LVEF. So, sensitive cTnI level may represent a marker of risk stratification, since it can provide useful information of underlying LV DD.

Consequently, the diagnosis of HFpEF in these patients should be supported by several measurement obtained from echocardiography, at rest or during exercise, blood test to evaluate NPs and sensitive cTnI levels, and, in case of uncertainty, invasive hemodynamic approach in order to exclude other causes (e.g., primary pulmonary hypertension, high output from arteriovenous shunting, lung disease, and obesity).

### Cardiac Amyloidosis

One of the specific etiologies of HFpEF is represented by cardiac amyloid deposition (165, 166). The most common forms of cardiac amyloidosis are light chain immunoglobulin amyloidosis (AL) and ATTR (167). Moreover, ATTR amyloidosis can be classified into two groups: the wild-type ATTR amyloidosis and the hereditary ATTR amyloidosis. It is often underdiagnosed because there are frequently alternative explanations for wall thickening and DD (e.g., arterial hypertension), but the recent discovery of therapeutical options for ATTR amyloidosis highlights the importance of identifying the specific etiology of HFpEF.

Wild-type ATTR amyloidosis seems to affect mainly male patient over 60 years of age. It has been identified in 13% of patients >60 years old with HFpEF and it was prevalent in 14–16% of older patients with severe calcified aortic stenosis undergoing transcatheter aortic valve replacement (166, 168–171). The clinical suspicion of cardiac amyloidosis should arise when the patient presents electrocardiographic anomalies (such as low QRS voltage) and bilateral carpal tunnel syndrome, signs and symptoms of HF, septal and posterior wall thickness >14 mm (i.e., Interventricular septum diameter (IVSd) and Posterior wall diameter (PWd) >14 mm with reduced GLS and apical sparing as optional criteria) at echocardiography, and age >65 years (172). The diagnosis of AL amyloidosis can be confirmed by abnormal hematological tests, cardiac imaging, and endomyocardial or extra-cardiac biopsy (Figures 1, 3). In case of suspicion of ATTR amyloidosis, bone tracer scintigraphy with planar and SPECT imaging represents an excellent diagnostic test, since it has a specificity and positive predictive value close to 100%. The endomyocardial biopsy remains the gold standard for the diagnosis (173).

Particular attention must be needed in case of patient with long-term dialysis: these patients have commonly IVSd and PWd >14 mm and systemic amyloidosis due to β2-microglobulin can involve the heart. Dialysis-related cardiac β2-microglobulin amyloidosis often occurs in patients who had undergone dialysis for 9 or more years with traditional low-flow dialysis membranes (174).

### Hypertrophic Cardiomyopathy

Among cardiomyopathies, HCM represents a specific etiology of HFpEF. It is diagnosed in case of increased myocardial wall thickness in the absence of abnormal loading conditions (Figure 4). In the majority of patients, HCM is caused by autosomal dominant sarcomere gene mutations. Hereditary syndromes, neuromuscular disorders, and storage disease (such as Anderson-Fabry disease) represent potential etiologies (22, 175). HFpEF is often diagnosed in patients affected by HCM and only at the end of the natural history of patients with HCM develop HFrEF (176, 177). Moreover, tachyarrhythmia (e.g., AF), ischemia, and acute or worsening mitral regurgitation or comorbidity can cause acute HF.

Therefore, the identification of HCM plays a pivotal role in the diagnostic process of HFpEF due to the different treatment options that need to be considered, both choice of drugs and risk assessment for sudden cardiac death.

### Severe CAD

Coronary artery disease is the most common cause of HF in industrialized countries. Patients with HFpEF or HFmrEF and CAD seem to have a worse cardiovascular prognosis as compared to patients with healthy coronary vessels (178, 179). On parallel, a complete coronary revascularization following the diagnosis of severe CAD is associated with improved survival in this subset of patients (180). Consequently, the screening for CAD in patients affected by HFpEF needs a careful assessment independently on the observed LVEF because of different outcome and response to treatment. Often, symptoms and non-invasive diagnostic stress testing seem to have inadequate predictive value in this group of patients, therefore coronary angiography may be appropriate.

Recently, after the publication of illuminating studies, such as the ISCHEMIA trial, there has been a lot of attention on new information derived by CCTA applications (181). In particular, high relevance is given to the identification of prognostic markers including, for instance, increased high-risk plaque volume and inflammatory activity around coronary arteries (e.g., fat attenuation index) (182–187). This change of paradigm inevitably leads to modify the therapeutic decisional processes (188).

## Conclusion

Heart failure with preserved ejection fraction represents a challenging clinical syndrome despite the fact that considerable progress is being made in its overall framing. The diagnosis of HFpEF relies mainly in the determination of elevated LV filling pressures, which can be non-invasively determined using a conventional transthoracic echocardiography in line with ESC guidelines statements. It is pivotal to follow an appropriate diagnostic pathway starting from the presence of the clinical suspicion, at first blood examinations and NP and stepping forward with morphological and functional diagnostic tools. Importantly, the interplay of advanced multi-modality imaging techniques plays a key role in screening and detection of specific etiologies that is essential step for evaluating tailored therapy.

## Author Contributions

All authors made a substantial contribution to the conception, design of this review paper, including drafting and revisions, and granted their approval for all aspects of the manuscript and its submission.

## Conflict of Interest

The authors declare that the research was conducted in the absence of any commercial or financial relationships that could be construed as a potential conflict of interest.

## Publisher's Note

All claims expressed in this article are solely those of the authors and do not necessarily represent those of their affiliated organizations, or those of the publisher, the editors and the reviewers. Any product that may be evaluated in this article, or claim that may be made by its manufacturer, is not guaranteed or endorsed by the publisher.
